# The Alga *Uronema belkae* Has Two Structural Types of [FeFe]-Hydrogenases with Different Biochemical Properties

**DOI:** 10.3390/ijms242417311

**Published:** 2023-12-09

**Authors:** Ghazal Alavi, Vera Engelbrecht, Anja Hemschemeier, Thomas Happe

**Affiliations:** Faculty of Biology and Biotechnology, Photobiotechnology, Ruhr University Bochum, 44801 Bochum, Germany; ghazal.alavi@ruhr-uni-bochum.de (G.A.); vera.engelbrecht@ruhr-uni-bochum.de (V.E.)

**Keywords:** [FeFe]-hydrogenases, hydrogen metabolism, microalgae, recombinant hydrogenases, *Uronema belkae*

## Abstract

Several species of microalgae can convert light energy into molecular hydrogen (H_2_) by employing enzymes of early phylogenetic origin, [FeFe]-hydrogenases, coupled to the photosynthetic electron transport chain. Bacterial [FeFe]-hydrogenases consist of a conserved domain that harbors the active site cofactor, the H-domain, and an additional domain that binds electron-conducting FeS clusters, the F-domain. In contrast, most algal hydrogenases characterized so far have a structurally reduced, so-termed M1-type architecture, which consists only of the H-domain that interacts directly with photosynthetic ferredoxin PetF as an electron donor. To date, only a few algal species are known to contain bacterial-type [FeFe]-hydrogenases, and no M1-type enzymes have been identified in these species. Here, we show that the chlorophycean alga *Uronema belkae* possesses both bacterial-type and algal-type [FeFe]-hydrogenases. Both hydrogenase genes are transcribed, and the cells produce H_2_ under hypoxic conditions. The biochemical analyses show that the two enzymes show features typical for each of the two [FeFe]-hydrogenase types. Most notable in the physiological context is that the bacterial-type hydrogenase does not interact with PetF proteins, suggesting that the two enzymes are integrated differently into the alga’s metabolism.

## 1. Introduction

[FeFe]-hydrogenases, which occur in prokaryotes and unicellular eukaryotes, are biocatalysts with high H_2_ turnover rates [1,2,3]. The catalytic site of [FeFe]-hydrogenases, called the H-cluster, consists of two sub-clusters, a standard cubane [4Fe4S] cluster (4Fe_H_) and the catalytic diiron moiety (2Fe_H_), which are covalently coupled through a coordinating cysteine residue. The two Fe atoms of 2Fe_H_ are bridged by an azadithiolate (adt) ligand and coordinated by two CN^–^ and three CO ligands [4,5,6]. The H-cluster undergoes changes in redox states, enabling the reduction of protons or oxidation of H_2_, which requires the simultaneous transfer of both protons and electrons [7,8,9].

[FeFe]-hydrogenases can be categorized into different structural types termed M1 to M5, depending on the number and type of additional FeS clusters. The N-terminal accessory clusters in the so-termed F-domain facilitate electron transfers within the protein [10,11]. M1-type hydrogenases, found, for example, in the unicellular chlorophycean algal species *Chlamydomonas reinhardtii* [12] and *Scenedesmus obliquus* [13], consist only of the protein domain harboring the H-cluster, the so-termed H-domain. All bacterial [FeFe]-hydrogenases known to date contain additional domains and FeS clusters. CpI, a well-studied [FeFe]-hydrogenase from *Clostridium pasteurianum*, is an M3-type hydrogenase that possesses both the H-domain and an F-domain with four accessory FeS clusters [4]. The two accessory clusters near the H-cluster, FS4A and FS4B, resemble [4Fe4S] clusters of bacterial-type ferredoxins. Two additional accessory clusters, FS2 and FS4C, are located near the protein surface. FS2 represents a [2Fe2S] cluster similar to that in plant-type ferredoxins, while FS4C is a [4Fe4S] cluster coordinated by three cysteines and one histidine residue [14,15,16]. In M3-type hydrogenases like CpI, electrons are transferred through the accessory FeS clusters in a sequential manner, facilitating long-range electron transport between the H-cluster and external redox partners [11,17,18].

About eighty years ago, it was discovered that several species of unicellular microalgae develop a H_2_ metabolism under hypoxic conditions, which is often coupled to photosynthesis [19,20,21]. Today, it is understood that algal H_2_ production often serves the function of dissipating excess electrons during fermentation [22,23] as well as during photosynthesis. In the latter case, electrons are transferred to the hydrogenases when other electron sinks such as the Calvin–Benson–Bassham (CBB) cycle are inactive, which happens transiently after a dark–light shift of hypoxic algal suspensions or in the time-scale of days upon nutrient deprivation (see [24,25] and references therein). The function of H_2_ oxidation is much less understood, but early studies showed that microalgae can reduce CO_2_ with the help of H_2_ oxidation [19,26,27]. The first algal hydrogenases that were characterized on the genetic and protein level were from the chlorophycean clade, such as CrHydA1 from *C. reinhardtii* [12,13,28,29]. These hydrogenases were all of the M1-type [30] and are very O_2_-sensitive [31]. Despite their intolerance towards O_2_, many of the hydrogenases of chlorophycean microalgae were shown to be linked to the photosynthetic electron transport chain through photosynthetic ferredoxin PetF as their natural electron donor [13,29,32,33]. While the precise evolutionary origin of algal hydrogenases is unclear, it is assumed that algae received their hydrogenase genes very early in evolution, and that the ancient hydrogenase gene encoded a hydrogenase with an F-domain [24,34]. The discovery of an M3-type [FeFe]-hydrogenase (CvHydA1) in the trebouxiophycean alga *Chlorella variabilis* NC64A [34] suggested that M1-type hydrogenases might have exclusively evolved in the chlorophycean clade, and the possession of larger hydrogenases was attributed to more ancient algal lineages like the Trebouxiophyceae, Rhodophytes, and Glaucophytes [34,35]. Notably, the M3-type enzyme CvHydA1 displays characteristics that distinguish it both from standard bacterial M3-type and chlorophycean M1-type [FeFe]-hydrogenases. The clostridial M3-type hydrogenase CpI is quite promiscuous regarding its redox partners, accepting electrons from many bacterial and plant-type ferredoxins [36,37]. In contrast, Engelbrecht et al. (2017) demonstrated that CvHydA1 did not interact with plant-type ferredoxins in vitro [37]. However, H_2_ production in *C. variabilis* NC64A is clearly light-dependent [37] and inhibited by the specific photosystem II (PSII) inhibitor 3-(3,4-dichlorophenyl)-1,1-dimethylurea (DCMU) as well as the cytochrome *b*_6_*f* complex inhibitor 2,5-dibromo-3-methyl-6-isopropylbenzoquinone (DBMIB) [37], suggesting that CvHydA1 is similarly coupled to the photosynthetic electron transport chain as the chlorophycean M1-type [FeFe]-hydrogenases. Since plant-type ferredoxin PetF is the soluble electron acceptor of photosystem I (PSI) and, so far, the only known natural electron donor for algal hydrogen production, it remains an open question how photosynthetically provided low-potential electrons are transferred to CvHydA1. CvHydA1 also differs from CpI regarding its O_2_ tolerance: while the clostridial CpI enzyme is more resistant to O_2_ than the highly O_2_-susceptible M1-type algal [FeFe]-hydrogenase CrHydA1, the M3-type algal hydrogenase CvHydA1 is almost as sensitive as CrHydA1 [37].

With the aim of gaining more insights into algal M3-type hydrogenases, we screened the 1000 Plant (1KP) Transcriptomes [38,39] for additional candidates. Interestingly, we identified two hydrogenase sequences in the chlorophycean species *Uronema belkae*, one encoding an M3-type, and the other one an M1-type enzyme. To our knowledge, this is the first time that (i) an M3-type hydrogenase was identified in the chlorophycean clade, and (ii) two structurally different hydrogenase types are encoded in one algal species. We show that *U. belkae* is capable of H_2_ production, and that both hydrogenase genes are transcribed. The biochemical characterization of the recombinant enzymes revealed differences regarding their interaction with plant-type ferredoxins as well as in their catalytic bias and in their sensitivity towards oxygen. The presence of two structurally and catalytically different hydrogenases in the same species suggests that the enzymes have different metabolic functions.

## 2. Results

### 2.1. H_2_ Production of U. belkae Is Connected to Photosynthesis

By using known algal [FeFe]-hydrogenases as queries, we detected two sequences in the *U. belkae* transcriptome that encode for a potential M1-type and an M3-type [FeFe]-hydrogenase, which we termed UbHydA1 (M1-type) and UbHydA2 (M3-type) (see the Section 4 for details; the sequences are provided in the Appendix A, Sequences A1).

**Figure 1:**
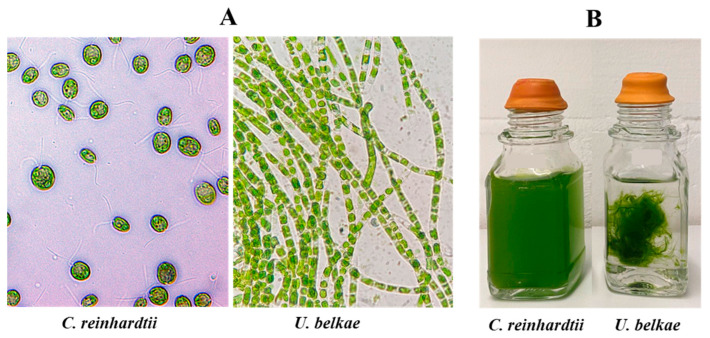
Visual comparison of *C. reinhardtii* and *U. belkae*. Panel (**A**) displays microscopic images of *C. reinhardtii* (**left**) and *U. belkae* (**right**). Note that the pictures are not shown to scale, but taken at 1000× (*C. reinhardtii*) or 400× (*U.* belkae) magnification. Panel (**B**) depicts both algal species in liquid media, with *C. reinhardtii* to the left and *U. belkae* to the right.

We first tested whether *U. belkae* develops a H_2_ metabolism under similar conditions as has been shown for other algae like *C. reinhardtii* and *S.* *obliquus*, namely under anaerobic conditions in the dark, after a dark–light shift of dark-anoxically incubated cells [24,25], as well as under sulfur deprivation [40]. To analyze whether H_2_ production of *U. belkae* in the light depends on electrons derived from the photosynthetic electron transport chain, the H_2_ production of cells shifted from dark-anoxia to illumination was also analyzed in the presence of the PSII inhibitor DCMU, the cytochrome *b*_6_*f* complex inhibitor DBMIB, and the proton gradient uncoupler CCCP. Routinely, we analyzed *C. reinhardtii* in parallel to ensure that the conditions were correctly applied. However, it must be noted that *U. belkae* exhibits a habitus very different from *C. reinhardtii* (Figure 1). While the latter usually forms a homogenous suspension of single cells, *U. belkae* forms filaments (Figure 1A) and large aggregates (Figure 1B). This not only resulted in our inability to extract exact amounts of cells from a given culture (forcing us to employ the dry weight of whole cultures for normalization), but very likely also to different responses of the cells, for example, due to a strong impact of self-shading in *U. belkae* cultures.

**Figure 2:**
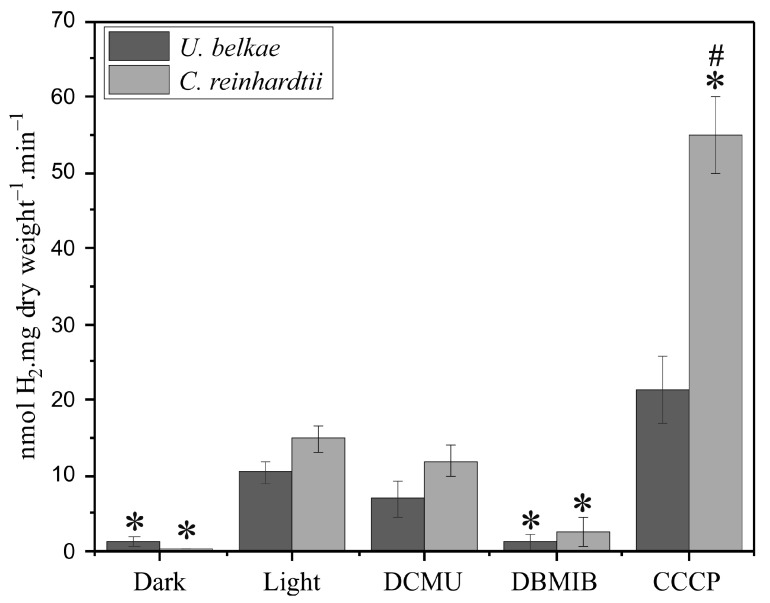
In vivo H_2_ production of *U. belkae* compared to that of *C. reinhardtii*. For each condition, one 120 mL sealed glass flask with 100 mL of culture was shaded and purged with argon for 90 min. Afterward, the argon flow was stopped, and the cultures were incubated for 15 min in continuous darkness or shifted to illumination. The latter condition was also applied in the presence of inhibitors of the photosynthetic electron transport chain (5 µM DCMU, 100 µM DBMIB, or 10 µM CCCP). After 15 min, H_2_ was quantified in the headspace. The dry weights of the whole cultures were determined afterward. *C. reinhardtii* cultures treated with the protocol were employed as experimental controls. The columns show the average of two biological replicates, analyzed in independent experiments, and error bars indicate the standard deviation. *T*-tests for independent samples were performed using Dell Statistica. Asterisks and hash signs denote a statistically significant (*p*-value ≤ 0.05) difference compared to rates observed in the light in the absence of inhibitors, and between species, respectively.

**Figure A1:**
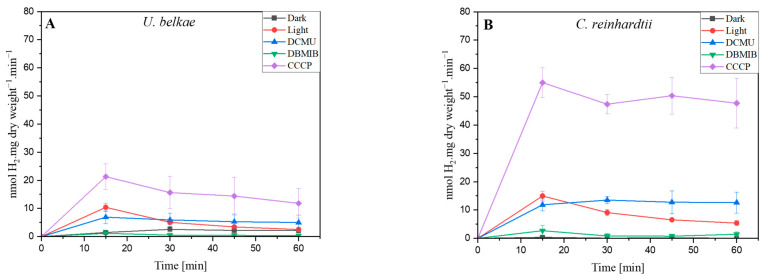
In vivo H_2_ production rates of *U. belkae* (**A**) compared to that of *C. reinhardtii* (**B**) over a time course of one hour. The data shown are from the same experiments of which the 15 min timepoints are shown in Figure 2, but extended to the 60 min timepoint. The rates shown were always calculated based on the net gain of H_2_ produced during the previous 15 min so that they represent the rates within a 15 min window. The columns show the average of two biological replicates, analyzed in technical triplicates in independent experiments, and the error bars indicate the standard deviation.

In vivo H_2_ evolution of *U. belkae* was detected under all conditions tested. Dark anoxia conditions were established by purging shaded algal cultures with argon for 90 min. During the set-up of these experiments, we ensured that this period was sufficient to induce in vitro hydrogenase activity in both *C. reinhardtii* and *U. belkae*. Subsequently, the argon flow was stopped, and the cells were exposed to different conditions, namely continued darkness, light, and light in the presence of the inhibitors stated above. In all cases, H_2_ was quantified in the headspace after 15 min (Figure 2) and then in 15 min intervals up to the 60 min timepoint (Appendix A; Figure A1). H_2_ production by *U. belkae* cultures was already observed during dark incubation, and upon exposure to light, the H_2_ production rate was about seven-fold higher than that of shaded cells (Figure 2). When the cells were exposed to light in the presence of inhibitors, a comparison between the conditions with and without inhibitors showed that the application of DCMU and, in particular, DBMIB led to a lower H_2_ production rate, while the use of CCCP resulted in increased H_2_ production (Figure 2). Overall, the same trends were observed for *C. reinhardtii*, although the extent of the differences between different treatments differed (Figure 2). Over a time course of 60 min, the trends described for the 15 min timepoint stayed similar, although the H_2_ production rates in the light declined moderately (Appendix A; Figure A1). These findings align with previous studies on algal strains, including *C. reinhardtii* [13,41,42,43], and suggest that photosynthetic electron transport is involved in providing electrons to the *U. belkae* hydrogenase(s).

**Figure A2:**
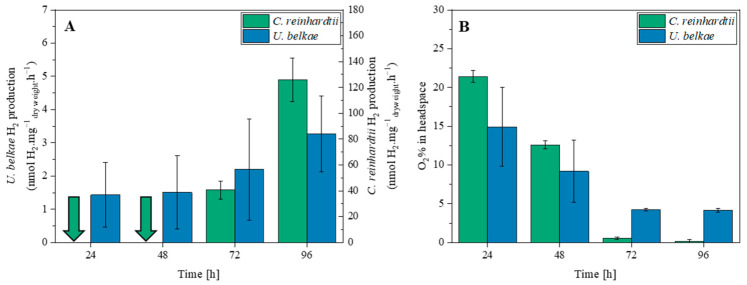
H_2_ production rates and O_2_ in the headspace of *U. belkae* and *C. reinhardtii* cultures under sulfur deprivation. (**A**) H_2_ production rates. Note the different y-axis scales: the primary and secondary y-axes correspond to *U. belkae* and *C. reinhardtii* H_2_ production rates, respectively. Green arrows indicate zero H_2_ production by *C. reinhardtii*. (**B**) O_2_ content in the headspace above the cultures. (**A**,**B**) Cells in their exponential growth phase were washed three times with TAP-S medium (TAP medium without any sulfur source) and then placed in 120 mL glass bottles, sealed, and positioned in front of a light source. Daily measurements of headspace gas were taken over a four-day period, starting at t = 0 h when no hydrogen was present. The columns display the average of two biological replicates, analyzed during independent experiments, with error bars indicating the standard deviation.

We also tested whether *U. belkae* would develop a H_2_ metabolism under sulfur (S) deficiency. In *C. reinhardtii*, the acclimation to S deprivation involves a down-regulation of CO_2_ assimilation and PSII activity, finally resulting in endogenously established hypoxia in sealed cultures in the light ([25] and references therein). Subjecting *U. belkae* to S deficiency indeed resulted in a gradual accumulation of H_2_ in the gas phase (Appendix A; Figure A2A), but the rates, calculated on a daily basis, were very low compared to what was achieved by *C. reinhardtii*, and also much lower than rates observed in argon-purged cells in full medium (compare Figure A2A in the Appendix A with Figure 2). The O_2_ content of the headspaces of both S-deprived algal cultures decreased. However, about 4% O_2_ remained in the headspace of *U. belkae* cultures, whereas almost no O_2_ was detectable above S-deficient *C. reinhardtii* cultures after 96 h (Appendix A; Figure A2B).

**Figure A3:**
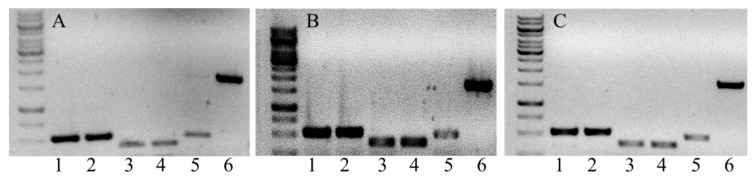
Gel electrophoresis analysis of products of PCR amplification of *U. belkae UbHydA1*, *UbHydA2*, and *RACK1* using genomic DNA or cDNA from (**A**) aerobically grown cells, (**B**) dark and anaerobically incubated cells, and (**C**) sulfur-deprived algae. (**A**–**C**) Lane 1: *UbHydA1* cDNA; lane 2: *UbHydA1* gDNA; lane 3: *UbHydA2* cDNA; lane 4: *UbHydA2* gDNA; lane 5: *UbRACK1* cDNA; and lane 6: *UbRACK1* gDNA. *RACK1* was used to validate the absence of gDNA within the RNA samples.

We employed RNA isolated from *U. belkae* cultures in which we had detected H_2_ production as well as cells grown under standard conditions to confirm that the genes encoding UbHydA1 and UbHydA2 were expressed in living algae. Both hydrogenase transcripts were indeed present under dark-anaerobic and sulfur deprivation conditions, but also under aerobic standard conditions (Appendix A; Figure A3).

### 2.2. Recombinant UbHydA1 and UbHydA2 Are Active [FeFe]-Hydrogenases

We then tested whether both putative [FeFe]-hydrogenases from *U. belkae* are active enzymes, and whether they have different biochemical features. Recombinant UbHydA1 and UbHydA2 were first analyzed regarding their catalytic activity in vitro using methyl viologen as the electron mediator. Well-characterized algal and bacterial [FeFe]-hydrogenases were used as controls, namely CrHydA1 (*C. reinhardtii* HydA1, M1-type) and CpI (an M3-type [FeFe]-hydrogenase from *C. pasteurianum*). UbHydA1 (M1-type) and UbHydA2 (M3-type) displayed specific H_2_ production activities of 990 ± 35 µmol H_2_ × mg^−1^ × min^−1^ and 2097 ± 261 µmol H_2_ × mg^−1^ × min^−1^, respectively, measured in biological duplicates. This ranks UbHydA1 activity in the range of CrHydA1 activity (999 ± 203 µmol H_2_ × mg^−1^ × min^−1^) and the activity of UbHydA2 in the range of CpI activity (2110 ± 258 µmol H_2_ × mg^−1^ × min^−1^), e.g., [18,44].

### 2.3. UbHydA1, but Not UbHydA2, Interacts with Photosynthetic Ferredoxin

**Figure 3:**
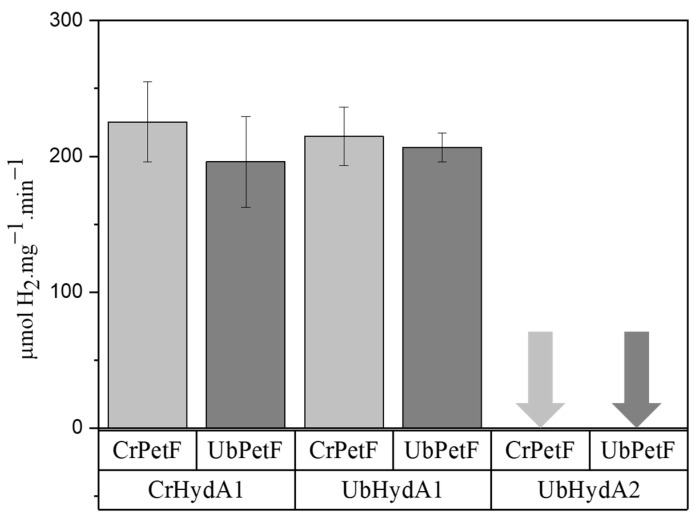
In vitro H_2_ production activities of recombinant *C. reinhardtii* CrHydA1, and *U. belkae* UbHydA1 and UbHydA2 with algal [2Fe2S]-ferredoxins as electron mediators (40 μM CrPetF from *C. reinhardtii*, 40 μM UbPetF from *U. belkae*). Sodium dithionite (NaDT) served as a reductant, and H_2_ production activities in the presence of NaDT only were subtracted from the rates measured with ferredoxins present. Error bars indicate the standard deviation for *n* = 2 biological replicates.

The effect of inhibitors on H_2_ photoproduction (Figure 2) suggested that one or both hydrogenases of *U. belkae* are connected to the photosynthetic electron transport chain. Photosynthetic ferredoxin PetF is known to be the physiological redox partner of algal hydrogenases, itself receiving electrons from PSI [33]. The only other M3-type hydrogenase from algal origin studied on the protein level, CvHydA1 from *C. variabilis* NC64A, cannot receive electrons from PetF or other plant type ferredoxins in vitro [37]. In contrast, the bacterial M3-type hydrogenase CpI is promiscuous and can accept electrons from PetF [36,37]. We therefore tested the H_2_ production activities of UbHydA1 and UbHydA2 with both *C. reinhardtii* and *U. belkae* PetF proteins (CrPetF and UbPetF, respectively) as the electron mediators. While the M1-type [FeFe]-hydrogenase UbHydA1 showed activities similar to those of CrHydA1 with both ferredoxins, UbHydA2 showed no PetF-driven activity (Figure 3).

### 2.4. Electrochemical Characterization of UbHydA1 and UbHydA2

**Figure 4:**
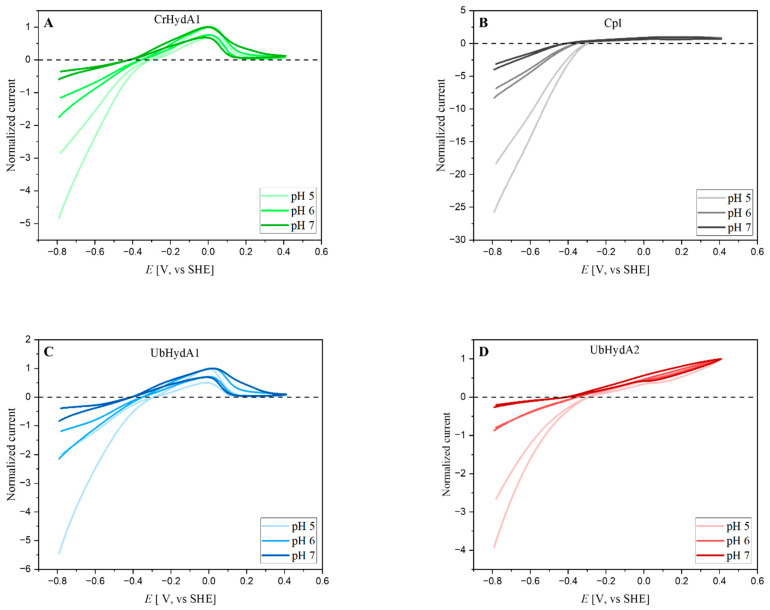
Cyclic voltammetry experiments of UbHydA1 and UbHydA2 in comparison to CrHydA1 and CpI. The currents recorded were all normalized to the respective H_2_ oxidation maxima for CrHydA1 ((**A**), green lines), CpI ((**B**), gray lines), UbHydA1 ((**C**), blue lines), and UbHydA2 ((**D**), red lines) at pH values of 5, 6, and 7. Experimental conditions were as follows: temperature = 10 °C, electrode rotation at 3000 rpm, 5 mV/s scan rate, measured in a mixed buffer system as described in the Section 4.

Hydrogenases are bidirectional enzymes that also catalyze the oxidation of hydrogen gas to protons and electrons [1]. To be independent from soluble electron mediators whose affinity to the enzymes might vary, we performed protein film electrochemistry (PFE) [45] to analyze the catalytic reversibility and the bias of the *U. belkae* hydrogenases. During PFE experiments, electric potentials are applied as driving forces for redox enzymes, and the resulting currents correspond to the catalytic activities. During cyclic voltammetry, the applied redox potential is increased and decreased again, and, in case of hydrogenases, negative currents represent H_2_ production, and positive currents reflect H_2_ uptake [46]. The cyclic voltammograms (CVs) of the electrocatalytic activities of UbHyA1 and UbHydA2 were recorded at three different pH values (pHs 5, 6, and 7), and were compared to the known behavior of CrHydA1 and CpI. UbHydA1 and UbHydA2 are clearly bidirectional enzymes like most hydrogenases analyzed to date [1]. Notable currents were recorded both at reducing and oxidizing potentials at all three pH values, and the voltammograms cut through the zero-current line at the potentials expected for the 2H^+^/H_2_ redox couple, namely at −0.295 V, −0.354 V, and −0.413 V vs. SHE at pH values of 5, 6, and 7, respectively (Figure 4).

UbHydA1, like CrHydA1, showed a slight inflection at the zero-current axis (Figure 4A,C), which suggests that a low overpotential is necessary for electron transfer to and from the H-cluster to occur. In contrast, UbHydA2 behaved similarly to CpI in that its CVs exhibited a sharp intersection at the zero-current axis (Figure 4B,D). The latter has been attributed to the presence of the additional iron–sulfur clusters that mediate electron exchange between the active site and the electrode surface [31,47,48]. At high potentials (>0 V vs. SHE), the H-domain only M1-type algal enzymes, UbHydA1 and CrHydA1, were inactivated; however, the decrease in H_2_ oxidation current recovered partially on the reverse scan. The inactivation at high potentials is termed anaerobic oxidative inactivation [49], and this inactivation was only moderate in the case of UbHydA2, comparable to the algal M3-type enzyme CvHydA1 [37], as well as to several bacterial M3-type [FeFe]-hydrogenases [50].

The currents determined during the CV experiments can be employed to calculate the catalytic bias of redox enzymes based on the currents at set positive and negative potentials around their standard redox potential. Table 1 shows that all hydrogenases analyzed here shifted towards a higher ratio of H_2_ oxidation: H_2_ production, determined at +250 mV and –250 mV relative to the respective standard redox potential, with increasing pH values, whereas the different hydrogenases exhibited varying degrees of bias. For instance, at pH 7, CrHydA1 exhibited a bias of 1.36 (Table 1), indicating a slightly higher rate of H_2_ oxidation compared to proton reduction, consistent with a previous study [44]. At this pH value, UbHydA1′s bias was determined to be 0.92, suggesting a nearly equal rate of H_2_ oxidation and evolution (Table 1). Notably, UbHydA2 displayed a more pronounced pH-dependent increase in bias, from 0.31 ± 0.01 (pH 5) to 1.74 ± 0.05 (pH 7) (Table 1). In contrast, CpI maintained a consistently low bias across all pH values (0.11 to 0.28), indicating its preference for H_2_ evolution. Indeed, at pH 5, CpI’s H_2_ evolution rate is nine times higher than its H_2_ oxidation rate [51].

### 2.5. Oxygen Sensitivity of UbHydA1 and UbHydA2

**Figure 5:**
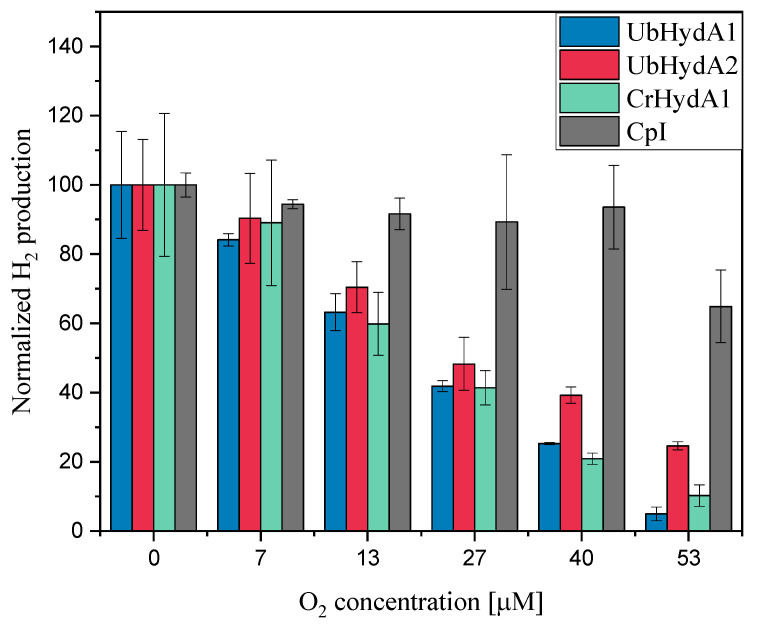
H_2_ production rates of recombinant [FeFe]-hydrogenases after O_2_ exposure. The [FeFe]-hydrogenases UbHydA1 (blue columns), UbHydA2 (red columns), CrHydA1 (green columns) and CpI (gray columns) were incubated for 5 min in the in vitro hydrogenase activity assay buffer in which different O_2_ concentrations had been adjusted by adding O_2_-saturated buffer. Following this incubation period, a mixture of NaDT and MV was added. NaDT served as both the reductant and to scavenge any remaining O_2_. The reaction mixtures were then incubated for 20 min at 37 °C, after which H_2_ in the headspace was quantified using gas chromatography. The columns show the average of two independent experiments, error bars indicate the standard deviation.

Most of the [FeFe]-hydrogenases studied to date are sensitive towards O_2_ [31,50,52,53,54]. Dioxygen binds to the open coordination site at the iron atom of the 2Fe_H_ subcluster distal to 4Fe_H_ (termed Fe_d_) resulting in irreversible structural damage to the H-cluster [52,55,56]. However, the reactivity of [FeFe]-hydrogenases to O_2_ varies [50,57,58]. To gain insights into the O_2_ sensitivity of the *U. belkae* [FeFe]-hydrogenases, we performed the standard in vitro hydrogenase assay except that the enzymes were pre-incubated in buffers with defined O_2_ concentrations (Figure 5). UbHydA1 exhibited a high sensitivity to O_2_, comparable to CrHydA1, in that its activity decreased sharply with increasing O_2_ concentrations. After a 5 min incubation in 53 µM O_2_, hardly any activity was left (Figure 5). Although UbHydA2 showed a slightly higher O_2_ stability compared to UbHydA1 and CrHydA1, retaining about 25% of activity after incubation in 53 µM O_2_, its stability was significantly lower than that of CpI, which was hardly affected by O_2_ concentrations up to 40 µM and still showed about 60% of activity after a 5 min treatment with 53 µM O_2_ (Figure 5), similar to previous results [31,37].

## 3. Discussion

To broaden the knowledge on algal [FeFe]-hydrogenases, particularly on the just recently analyzed M3-type algal enzymes, we made use of the strongly increased number of genomes and whole-genome transcriptomes to search for [FeFe]-hydrogenases in diverse algal species. In contrast to previous studies that suggested that algae have either H-domain-only M1-type or F-domain-containing M3-type [FeFe]-hydrogenases, we detected both hydrogenase types encoded in the transcriptome of the filamentous alga *U. belkae*. Testing first for physiological H_2_ production, we show here that this species develops a H_2_ metabolism under hypoxic conditions similar to that of the well-studied alga *C. reinhardtii* (Figure 2; Appendix A; Figure A1). *C. reinhardtii* and other algae produce low amounts of H_2_ in the dark, whereas H_2_ production is strongly accelerated after a dark–light shift ([25,59,60] and references therein). Photosynthetic H_2_ production is strictly dependent on electrons provided by the cytochrome *b*_6_*f* complex and PSI [13,37,43,61], whereas the extent of contribution of electrons provided by PSII differs [21,62]. Uncouplers of the proton gradient such as CCCP or FCCP (carbonyl cyanide *p*-trifluoromethoxyphenylhydrazone) usually result in enhanced H_2_ photoproduction rates [21,41]. This behavior was recapitulated here for both *U. belkae* and *C. reinhardtii* (Figure 2; Appendix A; Figure A1). Our comparisons of shaded and illuminated *U. belkae* cultures, as well as the application of photosynthetic inhibitors, indicate that (one of) the hydrogenase(s) of *U. belkae* receive(s) electrons from the photosynthetic electron transport chain through similar mechanisms as those in other algal species: H_2_ production was notably stimulated by light, indicating that light-dependent processes provide electrons. The DCMU treatment resulted in only a minor decrease in the rates observed in the light, suggesting that a process termed ‘indirect photolysis’ [63] contributes electrons, namely a transfer of electrons to the plastoquinone pool that are not directly derived from water-oxidation, but from the oxidation of organic substrates [64]. In contrast to DCMU, the DBMIB treatment of illuminated, H_2_-producing *U. belkae* cultures resulted in H_2_ production rates about as low as those measured in the dark, showing that the photosynthetic electron transport chain downstream of the cytochrome *b*_6_*f* complex is required for light-dependent H_2_ production. Finally, the uncoupler CCCP resulted in much higher H_2_ generation rates, indicating that a proton-pumping electron transport chain is involved in H_2_ photoproduction by *U. belkae*. In contrast to these experiments, sulfur-deprived *U. belkae* cultures only produced very low amounts of H_2_ (Appendix A; Figure A2A). This suggests that the processes that lead to strong H_2_ generation in S-deficient *C. reinhardtii* cells (recently reviewed in [25]) do not take place in *U. belkae*, similar to what was observed for the green alga *Scenedesmus obliquus* [65]. The very low H_2_ amounts detected in *U. belkae* cultures incubated in S-free medium might well have just accumulated because of self-shading within the dense cell aggregates (Figure 1B). The O_2_ content of the headspace remained notably higher in the case of *U. belkae* compared to *C. reinhardtii* cultures, which might have impeded the development of high hydrogenase activities, because both *U. belkae* [FeFe]-hydrogenases are very O_2_-sensitive (Figure 5; and see below).

**Figure A4:**
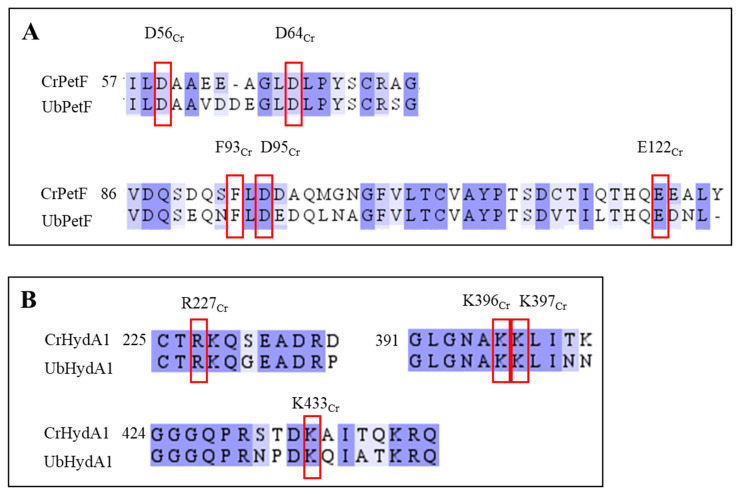
Partial amino acid sequence alignments of (**A**) *C. reinhardtii* and *U. belkae* PetF (CrPetF and UbPetF) and (**B**) *C. reinhardtii* and *U. belkae* HydA1 (CrHydA1 and UbHydA1). Alignments were performed in Jalview 2.11.2.7. A blue background indicates sequence parts with a conservation level of at least 50%. Positions marked with a red box present PetF residues (panel (**A**)) and HydA1 residues (panel (**B**)) with an influence on PetF-HydA activity [33,71,72]. Their respective positions in PetF and HydA1 of *C. reinhardtii* are indicated above the alignments.

In *C. reinhardtii*, photosynthetic ferredoxin PetF transfers electrons from PSI to its hydrogenases [33,44]. In this green alga, eleven additional ferredoxin isoforms have been detected [66], of which only FDX2 can efficiently donate electrons to CrHydA1 in vitro, although H_2_ production with FDX2 is usually lower than with PetF [67,68,69,70]. To date, no additional natural electron carriers are known that could mediate algal photosynthetic H_2_ production in vivo. We therefore explored whether the *U. belkae* hydrogenases would be able to accept electrons from *C. reinhardtii* PetF (CrPetF) and/or *U. belkae* PetF (UbPetF). The UbPetF sequence discovered in CNGBdb [38,39] shared a sequence identity of 70% with CrPetF, and all of the amino acids necessary for the interaction of CrHydA1 and CrPetF as based on the information provided by Winkler et al. (2009) are present (Appendix A; Figure A4A) [33]. Conversely, the amino acids present in CrHydA1 required for the interaction with CrPetF are also present in UbHydA1 (Appendix A; Figure A4B) [71,72]. Indeed, the M1-type [FeFe]-hydrogenase from *U. belkae*, UbHydA1, was capable of accepting electrons from both NaDT-reduced PetF proteins, the one from *C. reinhardtii* and the putative PetF from *U. belkae* (Figure 3). In contrast, *U. belkae’*s M3-type hydrogenase UbHydA2 showed no H_2_ production with either PetF protein. In this regard, UbHydA2 is similar to the algal M3-type [FeFe]-hydrogenase from *Chlorella variabilis* NC64A [37]. To date, the molecular basis for the inability of CvHydA1 to receive electrons from plant-type ferredoxins is not known, although an unfavorable surface charge has been suggested to contribute to it [37]. From the physiological point of view, it is interesting that both algal M3-type hydrogenases UbHydA2 and CvHydA1 cannot interact with plant-type ferredoxins in vitro, suggesting a specialized electron delivery system and, perhaps, their integration into a dedicated pathway. Since both *U. belkae* hydrogenase transcripts could be detected in cell samples from all conditions tested (aerobic standard growth, hypoxia, S deficiency) (Appendix A; Figure A3), we cannot speculate about one hydrogenase being more important under a certain condition.

Our electrochemical experiments suggest that the [FeFe]-hydrogenases from *U. belkae* display differences in their preference to reduce protons or to oxidize H_2_ (Table 1). Interestingly, at pH values of 6 and 7, UbHydA2 displayed a higher preference towards H_2_ consumption compared to UbHydA1, and at pH 7, UbHydA2 showed a notably higher preference for H_2_ oxidation than all of the other enzymes tested here (Table 1). As has been discussed for the two hydrogenase isoforms present in *C. reinhardtii* (CrHydA1 and CrHydA2), which have different preferences to reduce protons or to oxidize molecular hydrogen in vitro [44], this might suggest that UbHydA2 is involved in algal H_2_ consumption, and might not be employed for H_2_ production. Compared to the M3-type hydrogenase CpI, UbHydA2 is much more O_2_-sensitive (Figure 5), which is again similar to what was observed for the M3-type hydrogenase CvHydA1 from *C. variabilis* NC64A [37]. Both M3-type algal hydrogenases are almost as O_2_ sensitive as the algal M1-type enzymes (Figure 5) [37], which also excludes forming a robust hypothesis about any involvement in a H_2_ metabolism that may take place under a higher O_2_ pressure.

In summary, we identified an alga whose genome codes for two types of [FeFe]-hydrogenases—an M1- and an M3-type—that each, on the protein level, share similarities with the respective algal enzymes analyzed previously, namely CrHydA1, an M1-type hydrogenase found in *C. reinhardtii*, and CvHydA1, an M3-type hydrogenase found in *C. variabilis* NC64A. However, both enzymes appear to be present in one algal species, which, to our knowledge, has not been described before. The species *U. belkae* clearly has a H_2_ metabolism, and its similarity to that of *C. reinhardtii* suggests that *U. belkae* might also employ its hydrogenase(s) to prime photosynthesis after dark-hypoxic conditions as has been shown for *C. reinhardtii* (reviewed in [25]). However, the presence of two different hydrogenase types that are both active in their recombinant form suggests dedicated functions for each enzyme. This hypothesis is supported by the different behaviors of the enzymes in vitro which indicate that UbHydA2 (M3-type) cannot exchange electrons with plant-type ferredoxins and might prefer H_2_ oxidation vs. proton reduction. Elucidating the physiological role of M3-type [FeFe]-hydrogenases in algae as well as the structural features that makes them different from bacterial M3-type hydrogenases such as CpI might shed light on the evolution of algal [FeFe]-hydrogenases and H_2_ metabolism.

## 4. Material and Methods

### 4.1. Identification of [FeFe]-Hydrogenase- and PetF-Encoding Genes in Sequence Databases

The China National GeneBank (CNGBdb) was used to identify putative M1- and M3-type [FeFe]-hydrogenase sequences using the TBLASTN tool and *C. reinhardtii* (M1-type hydrogenase, GeneBank: AAL23572.1) and *C. variabilis* (M3-type hydrogenase, GenBank: AEA34989.1) orthologues as queries. The sequences from *U. belkae* that encode potential M1- and an M3-type hydrogenases were retrieved from CNGBdb from the 1000 plant (1KP) transcriptome database [38,39]. Here, we term these hydrogenases UbHydA1 (M1-type; ID: gnl|onekp|RAWF_scaffold_2042946) and UbHydA2 (M3-type; ID: gnl|onekp|RAWF_scaffold_2005753). The *U. belkae* PetF-encoding sequence (ID: gnl|onekp|RAWF_scaffold_2041009) was found in CNGBdb using *C. reinhardtii* PetF (GenBank: AAA33085.1) as the query sequence. The deduced protein sequences are provided in the Appendix A; Sequences A1.

### 4.2. Algal Strains, Growth Conditions and Induction of H_2_ Production

*U. belkae* wild-type SAG 34.86 and *C. reinhardtii* strain CC-124 wild-type cultures were grown in TAP (Tris–Acetate–Phosphate) medium [73]. Batch cultures were aerated with air containing 5% CO_2_ at room temperature under continuous illumination with Osram Lumilux CoolWhite light bulbs (Munich, Germany, 100 µmol photons × m^−2^ × s^−1^). To determine H_2_ production rates in the light and in the dark, cells were harvested by centrifugation (2 min, 2500× *g*, room temperature) in the mid-exponential stage of growth and subsequently resuspended in fresh TAP medium. A 100 mL volume of the cell suspensions was then transferred to shaded 120 mL flasks, sealed with gas-tight septa (red rubber Suba seals 37, Sigma–Aldrich, Taufkirchen, Germany, www.sigmaaldrich.com/DE/de, accessed on 1 December 2023), and continuously flushed with argon for 90 min. The presence of active [FeFe]-hydrogenases after this anaerobic incubation was evaluated by measuring the in vitro hydrogenase activity, using a method described previously [74]. The assay involved a reaction mixture consisting of 1% (*v*/*v*) Triton-X 100, a gentle detergent for algal cell lysis, along with 10 mM methyl viologen (MV) as an artificial electron mediator and 100 mM sodium dithionite (NaDT) as a reductant. To determine H_2_ production in vivo, the argon-purging was stopped, and the anaerobic algal cultures were either kept in darkness or were transferred to light (140 µmol photons × m^−2^ × s^−1^) with continuous shaking.

To analyze the connection between in vivo H_2_ production and the photosynthetic electron transport chain, inhibitors of the latter were applied: 5 µM DCMU (3-(3,4-dichlorophenyl)-1,1-dimethylurea), 100 µM DBMIB (dibromothymoquinone), or 10 µM CCCP (carbonyl cyanide *m*-chlorophenylhrazone) were added to the algal cultures individually.

To determine whether *U. belkae* establishes a H_2_ metabolism under sulfur deficiency, cells were harvested as described above, washed three times with TAP-S medium (TAP medium in which all sulfate components were replaced by chloride salts) and resuspended in TAP-S medium. A 100 mL volume of the algal culture was placed into sealed 120 mL glass flasks as described above and then incubated in continuous light (140 µmol photons × m^−2^ × s^−1^) at 20 °C with constant shaking.

In all cases, the H_2_ present in the headspace of reaction vessels or algal cultures was quantified using a gas chromatograph, model GC-2010 from Shimadzu (www.shimadzu.com, accessed on 1 December 2023), equipped with a thermal conductivity detector and a PLOT fused silica coating molsieve column (5 Å, 10 m by 0.32 mm, from Varian), employing argon as the carrier gas. The concentration of molecular oxygen (O_2_) in the headspace of algal cultures was determined employing the same GC set-up.

The dry weight of the algae was determined by pelleting the cells in 50 mL conical tubes pre-dried for 24 h, removing the supernatant, and subsequently incubating the cells at 120 °C for six hours to allow for complete drying.

### 4.3. DNA and RNA Analysis

To obtain RNA and DNA samples, algal cells were harvested by centrifugation and genomic DNA (gDNA) was purified by phenol/chloroform extraction, chloroform extraction, and ethanol precipitation according to standard procedures. Total RNA was isolated employing the NucleoSpin kit from Macherey-Nagel (Düren, Germany, www.mn-net.com, accessed on 1 December 2023) following the manufacturer’s instructions. After extraction, the DNase-treated RNA was immediately used for cDNA synthesis using Promega M-MLV Reverse Transcriptase (M1705, 200 U × µL^−1^; https://worldwide.promega.com, accessed on 1 December 2023), dNTPs, oligo(dT)_18_ primers, and RNase inhibitor (RNasin, N251, Promega, Madison, WI, USA). Thereafter, specific primers for the sequences coding for UbHydA1 (5′-CAGTGATTGCGGGCAGTTGGC-3′(F), 5′-GCTCCTCCAGAGCTGGAACAATG-3′ (R)) and UbHydA2 (5′- GCACATGCTGCTGATGG-3′ (F) and 5′-CAGTTATCAGCCTGCCTTG-3′ (R)) were employed in polymerase chain reactions (PCRs) employing Q5 High-Fidelity DNA polymerase. The same oligonucleotides were used in PCRs with gDNA as the template.

The *RACK1* gene from *C. reinhardtii*, which encodes receptor of activated protein kinase C (GenBank: CAA37638.1), is commonly used as a constitutively transcribed reference gene [75]. To obtain oligonucleotides for the *RACK1* gene of *U. belkae*, RACK1 from *C. reinhardtii* was used as a query in CNGBdb [38,39] to identify the *U. belkae* sequence. In addition to serving as a control for the presence and quality of RNA, we purposefully selected oligonucleotides that generated larger fragments from gDNA, and thus very likely spanned introns, allowing us to ensure the absence of gDNA from RNA samples. The oligonucleotides used for *RACK1* analysis were 5′-TGTATGAGGCCACTGTGAG-3′ (F) and 5′-CCACACTGGCTACATCAAC-3′ (R).

### 4.4. Recombinant Protein Production and Purification

Sequences encoding the hydrogenases and PetF from *U. belkae* were codon-optimized for heterologous expression in *Escherichia coli* and synthesized by Thermo Fisher Scientific (Waltham, MA, USA, www.thermofisher.com/, accessed on 1 December 2023). The coding sequences are provided in the Appendix A; Sequences A2.

*E. coli* strain BL21 (DE3) Δ*iscR* [76] was used for the heterologous expression of hydrogenase and ferredoxin sequences using the vector systems pET21b(+) (hydrogenases) and pASK-IBA7 (PetF). Expression and purification were performed as described earlier [44,77]. For the oxygen-sensitive hydrogenases, all steps were performed under strictly anoxic conditions. Briefly, the electrocompetent cells transformed with the specific plasmid were incubated at 37 °C until an OD_600_ of 0.35–0.5 was reached. For the production of hydrogenases, cultures were then transferred to an anoxic glove box with a N_2_:H_2_ (99:1) atmosphere. Expression was initiated by adding 0.1 mM IPTG (isopropyl β-D-1-thiogalactopyranoside) for hydrogenase-, and 0.2 µg × mL^−1^ AHT (anhydrotetracycline) for PetF-encoding sequences. After induction, the cell cultures were incubated overnight at room temperature while stirring at 130 rpm. Cells were harvested after 17–20 h by centrifugation. The recombinant Strep-tagged proteins were purified (anoxically) using Strep-Tactin Superflow high-capacity cartridges (IBA Lifesciences, Göttingen, Germany, www.iba-lifesciences.com, accessed on 1 December 2023) according to the manufacturer’s instructions. Proteins were eluted with 2.5 mM desthiobiotin in 100 mM Tris–HCl (Tris(hydroxymethyl)-aminomethan) buffer pH 8 and, in case of the hydrogenases, the buffer was supplemented with 2 mM NaDT, removing any residual oxygen. The purity of the proteins was examined by SDS–PAGE (sodium dodecyl sulfate polyacrylamide gel electrophoresis) and the protein concentration was determined using the Bradford method (Bio-Rad, Feldkirchen, Germany; www.bio-rad.com/, accessed on 1 December 2023) and bovine serum albumin (Biolabs, Heidelberg, Germany; www.biolabs.io/, accessed on 1 December 2023) as a standard. All proteins were stored at −80 °C until further use.

### 4.5. Determination of H_2_ Production Activity of Recombinant Hydrogenases

[FeFe]-hydrogenases heterologously produced in *E. coli* do only contain the [4Fe4S] part of the H-cluster [78] and are generally termed ‘apo’-hydrogenases. To obtain the active holo-proteins, they were maturated in vitro with a 10-fold excess of a chemically synthesized [2Fe_H_] cofactor mimic as described before [79]. In vitro hydrogen production of 400 ng holo-protein was measured in 2 mL reaction assays containing 100 mM NaDT as a sacrificial electron donor and 10 mM MV as an electron mediator in 100 mM potassium phosphate buffer, pH 6.8. After incubation at 37 °C for 20 min, H_2_ formation was analyzed by gas chromatography as described above.

The sensitivity of the [FeFe]-hydrogenases towards O_2_ was determined by incubating the enzymes in the same reaction mixture except that no NaDT and MV were included and that defined O_2_ concentrations were added in form of O_2_-saturated buffer. After an incubation of 5 min, a mixture of NaDT and MV was added to a final concentration of 200 mM and 10 mM, respectively. The in vitro hydrogenase activity assay was then conducted as described above.

To measure H_2_ production by the hydrogenases with ferredoxin as the electron mediator, 200 µL solutions containing 80 ng holo-hydrogenase, 25 mM NaDT, and 40 µM ferredoxin in 100 mM potassium phosphate buffer, pH 6.8, were used. The same assays but without ferredoxins served as background controls, and any H_2_ measured therein was subtracted from the values obtained from the ferredoxin-containing assays.

### 4.6. Protein Film Electrochemistry

Protein film electrochemistry was conducted in an anoxic glove box using a standard gastight three-electrode chemical cell, water-jacketed for temperature control as described before [44]. A rotating disk polypyrolytic graphite electrode (PGE) for protein adsorption, a Pt wire as the counter electrode, and Ag/AgCl as the reference electrode were utilized. The electrochemical cell contained a buffer mixture of 15 mM each MES, HEPES, TAPS, CHES, and sodium acetate supplemented with 0.1 M NaCl. For the measurements, 3 µL of a 10 µM protein solution were dropped onto the PGE and incubated for 5 min. The electrode was then rinsed with water to remove any non-adsorbed enzymes. The experiments were conducted at 10 °C, with a scan rate of 5 mV × s^−1^, in a 100% H_2_ atmosphere and a rotation rate of 3000 rpm. An Autolab potentiostat was used to control the potential. Cyclic voltammograms (CVs) were recorded between –0.8 and +0.4 V vs. SHE to analyze the catalytic behavior of the enzymes at different pH values. To determine the catalytic bias of each enzyme, CVs were recorded between −250 mV and +250 mV vs. their standard redox potential, and the respective endpoint currents at +250 mV and –250 mV were employed to calculate the ratio of H_2_ oxidation vs. H^+^ reduction.

## 5. Conclusions

Hydrogenases and H_2_ metabolisms of microalgae contribute to the plasticity of photosynthesis and fermentative pathways but may also be utilized for sustainable hydrogen production. Understanding the cellular pathways and functions of H_2_ production or oxidation, the enzymes responsible, as well as their evolutionary origins, will help us to understand, and perhaps apply, the physiology of these important primary producers. Previous work has already shown that microalgae exhibit differences regarding the conditions under which they produce or consume H_2_, and regarding the [FeFe]-hydrogenase-types responsible for these processes. This study contributes to the knowledge on microalgal H_2_ metabolism by adding a new species to the list of microalgal H_2_ producers, which, furthermore, has two different structural hydrogenase types. As our data suggest that the two [FeFe]-hydrogenases of *U. belkae* may be employed in different pathways, future studies may reveal previously unknown H_2_ metabolisms and their integration into the host’s physiology.

## Figures and Tables

**Table 1:** Catalytic bias of UbHydA1, CrHydA1, UbHydA2, and CpI as determined by cyclic voltammetry. Measurements were performed cycling ±250 mV around the equilibrium potentials at pH values of 5, 6, and 7, and the ratios of H_2_ oxidation versus H_2_ evolution were calculated by dividing the positive by the negative currents at +250 mV and –250 mV, respectively. Data shown represent the mean ± standard deviation for *n* = 2 biological replicates.

Enzyme	pH 5	pH 6	pH 7
UbHydA1	0.47 ± 0.01	0.58 ± 0.04	0.92 ± 0.02
CrHydA1	0.46 ± 0.02	0.7 ± 0.01	1.36 ± 0.06
UbHydA2	0.31 ± 0.01	0.72 ± 0.001	1.74 ± 0.05
CpI	0.11 ± 0.03	0.19 ± 0.01	0.28 ± 0.02

## Data Availability

The data presented in this study are available on request from the corresponding authors.

## Appendix A

### Appendix A.1. Sequences A1: Sequences of U. belkae Hydrogenases and PetF

>UbHydA1
MAVAEPKADCDCGPKAAGPHWQQALDLLDAKDKSKLFVVQIAPAVRVAISEPFGLPSGTITIGQIVTGLRQLGFDVVFDTLFGADLTIMEEGTELLHRLKDHLEGNPKNEEPLPMFTSCCPGWVELVEKSYPDMIPYLSSCKSPQMMLGAIIKNYFADVAGYAPQDVISCSVMPCTRKQGEADRPAGATTGLARDVDHVITTAELAKIFQDRGIDLPNLPESPLDNPIGEGSGAGQLFGTTGGVMEAAVRTVYELVTGQPMERINLTEVRGLDGIKEATLVLKPAPDSILGKWSGEGEGLPIRIAVANGLGNAKKLINNIKDGSAKYDFVEVMACPGGCISGGGQPRNPDKQIATKRQQSMYTIDERMTLRRSHDNVFIQQLYAKFLGKPGSHKAHDLLHTPYIPGGPAKQ

>UbHydA2
MSDKAAAQPPNQRRSLHNLHVPQNSTPRTRAAAHLLIPFRKQHAATTPRQHNIAEAQPHACVTINGRSVPFSPGQTILQAAANAGVQIPSICYHPRLPKSPGTCRVCLVSVDGRMRPSCVTEAQAGQVVETDSEEVREHVRGQLALLRTNHPDDCMTCDVNGRCEFQRLITQYQVPLMPKTLRIQNHTHAQEIKGLEDFDASAKAAPLGLDEPPSNASSPPPTTPESCASITAAAAASANTTTLRHHETPGLVSHGVAISVDQDKCIKCGRCVTMCQEVQQMNVLGWVGRGQEAHVGVVDDAELQQSRCIECGQCVSICPVGALTEHSEWRQVMQLLRSKRKIMVVQTAPAVRVAIGEEVGLAPGAISTGQLVTALRQLGFDYVFDTNFSADLTIMEEGSELLQRLQHHWQHPDSPAHTPPHEDPRFKGAGSAGAPKAGHHTPGPLPMFTSCCPAWINEVERDRPELIPHLSTCKSPQGMMGAVVKSIWAKKMGLNPEDVVLVSVMPCTAKKHEARREELLPEQPQGDALTPADTTTSVYTSTSGDGITGGDRVVCQEPARDVDFVLTTRELGTMLRQMGIPLASLEAGDYDHPLAGGTGAAVLFGATGGVMEAALRTVAAVVGGAPLPRLEMEAIRGMAGVKEATLQLEGPGLPGGAKEVRVAVASGIGAAQQLLDKVQSGQVNYDFIEVMSCPGGCIGGGGQPKTKDPTAILKRMDATYTIDQDSTLRQSHENPDVQQLYKDALEAPGSHDAHKLLHTHYTDRSSEVKS

>UbPetF
MAYKVTLKTPDGDKTFECADDKYILDAAVDDEGLDLPYSCRSGGCCTCTGKIVSGTVDQSEQNFLDEDQLNAGFVLTCVAYPTSDVTILTHQEDNL

### Appendix A.2. Sequences A2: Codon-Optimized Sequences Encoding Hydrogenases and PetF for Expression in E. coli

>UbHydA1_Codon_Optimized
ATGGCAGTTGCAGAACCGAAAGCAGATTGTGATTGTGGTCCGAAAGCCGCAGGTCCGCATTGGCAGCAGGCACTGGATCTGCTGGATGCAAAAGATAAAAGCAAACTGTTTGTGGTTCAGATTGCACCGGCAGTTCGTGTTGCAATTAGCGAACCGTTTGGTCTGCCGAGCGGCACCATTACCATTGGTCAGATTGTTACCGGTCTGCGTCAGCTGGGTTTTGATGTTGTTTTTGATACCCTGTTTGGTGCCGATCTGACCATTATGGAAGAAGGCACCGAACTGCTGCATCGTCTGAAAGATCATCTGGAAGGTAATCCGAAAAATGAAGAACCGCTGCCGATGTTTACCAGCTGTTGTCCTGGTTGGGTTGAACTGGTTGAAAAAAGCTATCCTGATATGATTCCGTATCTGAGCAGCTGTAAAAGTCCGCAGATGATGCTGGGTGCAATCATCAAAAACTATTTTGCAGATGTTGCAGGTTATGCACCGCAGGATGTTATTAGCTGTAGCGTTATGCCGTGCACACGTAAACAGGGTGAAGCAGATCGTCCGGCAGGCGCAACCACCGGTCTGGCACGTGATGTTGATCATGTTATTACCACCGCAGAACTGGCAAAAATCTTTCAGGATCGTGGTATTGATCTGCCGAATCTGCCGGAAAGTCCGCTGGATAATCCGATTGGTGAAGGTAGCGGTGCAGGTCAGCTGTTTGGTACAACCGGTGGTGTTATGGAAGCAGCCGTTCGTACCGTTTATGAACTGGTGACGGGTCAGCCGATGGAACGTATTAATCTGACCGAAGTTCGTGGTCTGGATGGTATTAAAGAAGCAACCCTGGTTCTGAAACCGGCACCGGATAGCATTCTTGGTAAATGGTCAGGTGAAGGTGAAGGCCTGCCGATTCGTATTGCAGTTGCAAATGGTCTGGGTAATGCCAAAAAACTGATCAACAACATTAAAGACGGCAGCGCCAAATATGATTTTGTTGAAGTTATGGCATGTCCCGGTGGCTGTATTAGCGGTGGTGGTCAGCCTCGTAATCCGGATAAACAAATTGCAACCAAACGTCAGCAGAGCATGTATACCATTGATGAACGTATGACCCTGCGTCGTAGCCATGATAATGTTTTTATTCAGCAACTGTACGCCAAGTTTCTGGGTAAACCGGGTAGCCATAAAGCACATGATCTGCTGCATACCCCGTATATTCCAGGTGGTCCGGCAAAACAG

>UbHydA2_Codon_Optimized
ATGAGCGATAAAGCAGCAGCACAGCCTCCGAATCAGCGTCGTAGCCTGCATAATCTGCATGTTCCGCAGAATAGCACACCGCGTACACGTGCAGCAGCCCATCTGCTGATTCCGTTTCGTAAACAGCATGCAGCAACAACACCGCGTCAGCATAATATTGCCGAAGCACAGCCGCATGCATGTGTTACAATTAATGGTCGTAGCGTTCCGTTTAGTCCGGGTCAGACCATTCTGCAGGCAGCAGCAAATGCCGGTGTTCAGATTCCGAGCATTTGTTATCATCCGCGTCTGCCGAAAAGTCCGGGTACATGTCGTGTTTGTCTGGTTAGCGTTGATGGTCGCATGCGTCCGAGCTGTGTTACCGAAGCGCAGGCAGGTCAGGTTGTTGAAACCGATAGCGAAGAGGTTCGTGAACATGTGCGTGGTCAGCTGGCACTGCTGCGTACCAATCATCCTGATGACTGTATGACCTGTGATGTGAATGGTCGTTGTGAATTTCAGCGTCTGATTACCCAGTATCAGGTTCCGCTGATGCCGAAAACACTGCGTATTCAGAATCATACCCATGCGCAAGAAATTAAAGGCCTGGAAGATTTTGATGCAAGCGCAAAAGCAGCACCGCTGGGTTTAGATGAACCGCCTAGCAATGCAAGCAGTCCGCCTCCGACCACACCGGAAAGCTGTGCAAGCATTACCGCAGCAGCAGCCGCAAGCGCCAATACCACCACACTGCGTCATCATGAAACCCCTGGTCTGGTGAGCCATGGTGTTGCAATTAGCGTGGATCAGGATAAATGTATTAAATGCGGTCGTTGCGTTACCATGTGTCAAGAGGTGCAGCAGATGAATGTTTTAGGTTGGGTTGGTCGTGGTCAAGAGGCACATGTTGGTGTTGTGGATGATGCAGAACTGCAGCAGAGCCGTTGTATTGAATGTGGTCAGTGTGTTAGCATTTGTCCGGTTGGTGCACTGACCGAACATTCAGAATGGCGTCAGGTTATGCAGCTGCTGCGTTCAAAACGCAAAATTATGGTTGTTCAGACCGCACCGGCAGTTCGTGTTGCCATTGGTGAAGAAGTTGGTCTGGCACCGGGTGCGATTAGCACAGGCCAGCTGGTTACCGCACTGCGCCAGCTGGGTTTTGATTATGTTTTTGATACCAACTTCAGCGCAGATCTGACCATTATGGAAGAAGGTAGCGAACTGCTGCAGCGTTTACAGCATCATTGGCAGCATCCGGATAGTCCGGCACATACCCCTCCGCATGAAGATCCGCGTTTTAAAGGTGCAGGTAGCGCAGGCGCACCGAAAGCAGGTCATCATACACCGGGTCCGCTGCCGATGTTTACCAGCTGTTGTCCGGCATGGATTAATGAAGTTGAACGTGATCGTCCGGAACTGATTCCGCATCTGAGCACCTGTAAAAGTCCGCAGGGCATGATGGGTGCAGTTGTTAAAAGCATTTGGGCCAAAAAAATGGGTCTGAATCCGGAAGATGTTGTGCTGGTGAGCGTTATGCCGTGTACCGCAAAAAAACATGAAGCACGTCGTGAAGAACTGTTACCGGAACAGCCGCAGGGTGATGCACTGACACCGGCAGATACCACAACCAGCGTTTATACCAGCACCTCTGGTGATGGTATTACCGGTGGTGATCGTGTTGTTTGTCAAGAACCGGCACGTGATGTTGATTTTGTTCTGACCACACGTGAACTGGGCACCATGCTGCGTCAGATGGGTATTCCGCTGGCAAGCCTGGAAGCCGGTGATTATGATCATCCGTTAGCCGGTGGTACAGGTGCAGCCGTTCTGTTTGGTGCAACCGGTGGCGTTATGGAAGCAGCACTGCGTACCGTTGCCGCAGTTGTTGGTGGTGCACCGCTGCCTCGTCTGGAAATGGAAGCAATTCGTGGTATGGCAGGCGTTAAAGAAGCAACCCTGCAGCTGGAAGGTCCGGGTCTGCCTGGTGGTGCCAAAGAAGTTCGCGTTGCAGTTGCAAGCGGTATTGGTGCAGCCCAGCAACTGCTGGATAAAGTTCAGAGCGGTCAGGTGAACTATGATTTTATTGAAGTTATGAGCTGTCCCGGTGGTTGTATTGGTGGTGGTGGCCAGCCGAAAACCAAAGATCCGACCGCAATTCTGAAACGTATGGATGCAACCTATACCATCGATCAGGATTCAACCCTGCGTCAGTCACATGAAAATCCGGATGTACAGCAACTGTATAAAGATGCACTGGAAGCACCGGGTAGCCATGATGCACATAAACTGTTACATACCCATTACACCGATCGTAGCAGCGAAGTGAAAAGC

>UbPetF_Codon_Optimized
ATGGCATATAAAGTGACCCTGAAAACACCGGATGGTGATAAAACCTTTGAATGTGCCGATGACAAATATATCCTGGATGCAGCAGTTGATGATGAAGGTCTGGATCTGCCGTATAGCTGTCGTAGCGGTGGTTGTTGTACCTGTACCGGTAAAATTGTTAGCGGCACCGTTGATCAGAGCGAACAGAATTTTCTGGATGAAGATCAGCTGAATGCAGGTTTTGTTCTGACCTGTGTTGCATATCCGACCAGTGATGTTACCATTCTGACCCATCAAGAAGATAACCTG
